# Empathy and musical reward sensitivity in middle childhood: a psychometric validation of the Barcelona music reward questionnaire in children aged 7–9

**DOI:** 10.3389/fcogn.2026.1804916

**Published:** 2026-08-13

**Authors:** Maria Celeste Fasano, Maria Grazia Monaci, Gianni Nuti, Manuela Filippa

**Affiliations:** 1 Department of Psychology and Behavioural Sciences, Aarhus University, Aarhus, Denmark; 2 Department of Human and Social Sciences, University of Valle d'Aosta, Aosta, Italy; 3 Department of Psychology and Educational Sciences, Swiss Center for Affective Sciences, University of Geneva, Geneva, Switzerland

**Keywords:** BMRQ, emotional evocation, empathy, middle childhood, musical reward

## Abstract

**Introduction:**

Music is a highly rewarding experience, yet individuals differ substantially in how pleasurable they find music. In adults, sensitivity to musical reward has been consistently linked to socio-emotional traits, particularly empathy. However, little is known about whether this association is already present in middle childhood, before music engagement becomes increasingly tied to identity formation and autonomous, self-directed use during adolescence. The present study examined musical reward sensitivity in middle childhood and its relationship with empathy.

**Methods:**

A sample of 621 children aged 7–9 years completed a child-adapted Italian version of the Barcelona Music Reward Questionnaire (BMRQ-MC) and the Empathy Questionnaire for Children and Adolescents (EmQue-CA). Confirmatory factor analyses tested the factorial structure of the BMRQ-MC and its measurement invariance across gender. Correlational and regression analyses examined associations of musical reward sensitivity with empathy, musical engagement, and musical ability.

**Results:**

The original five-factor BMRQ model showed only partial support, with the Musical Seeking subscale displaying poor reliability and weak factor loadings. A four-factor model showed improved fit and measurement invariance across gender. Musical reward sensitivity was positively associated with empathy, particularly affective empathy, and showed a small positive association with musical engagement, whereas objective musical ability was unrelated.

**Discussion:**

These findings support the psychometric validity of the BMRQ-MC for assessing musical reward sensitivity in middle childhood and suggest that, at this developmental stage, individual differences in musical reward are more closely linked to socio-emotional dispositions and musical engagement than to perceptual musical skills. The study highlights empathy as a key correlate of early musical pleasure and positions music reward sensitivity as a socio-emotionally grounded individual difference relevant to children's developing social functioning.

## Introduction

Music is one of the most universal and rewarding human experiences, yet individuals differ widely in the degree to which they experience music as pleasurable (Gold et al., 2019; Mas-Herrero et al., 2013). Understanding the mechanisms underlying this variability has become a central aim in the study of music, emotion, and reward. The Barcelona Music Reward Questionnaire (BMRQ; Mas-Herrero et al., 2013) was developed to assess individual differences in music-related reward sensitivity across five theoretically grounded components: Emotional Evocation, Mood Regulation, Social Reward, Musical Seeking, and Sensory-Motor. Since its introduction, the BMRQ has been employed in studies linking musical reward sensitivity to a range of personal characteristics, including personality, emotional regulation, and social-cognitive traits (Martínez-Molina et al., 2016; Mas-Herrero et al., 2014; Wang et al., 2023). This body of work consistently shows that musical reward is not uniform, but shaped by relatively stable individual dispositions, supporting the notion that sensitivity to musical pleasure reflects enduring dispositional factors rather than transient states.

Among these dispositional characteristics, empathy has emerged as a particularly relevant construct (Carraturo et al., 2022; Kawakami and Katahira, 2015; Kreutz and Cui, 2022). Empathy is commonly defined as the capacity to share, understand, and respond to the emotional states of others, and contemporary models distinguish affective components of emotional sharing from cognitive components of perspective taking (Decety and Holvoet, 2021; Decety and Jackson, 2004). Music constitutes a temporally unfolding and emotionally expressive stimulus that can engage affective resonance and emotional contagion, processes through which perceived expressive cues are transformed into felt emotion (Juslin, 2013; Juslin and Västfjäll, 2008; Koelsch, 2014). These processes conceptually overlap with core components of empathic responding. Consistent with this view, adults high in trait empathy report stronger emotional responses to music, greater enjoyment of emotionally complex or negatively valenced music, and more frequent use of music for emotional self-regulation (Sachs et al., 2015; Taruffi and Koelsch, 2014; Vuoskoski et al., 2012). These findings suggest that empathy may be closely linked to individual differences in how rewarding music is experienced.

Despite robust evidence in adults, considerably less is known about when and how the association between empathy and musical reward becomes evident across development. To date, only very limited evidence is available in younger populations. Kawakami and Katahira (2015) showed that, in 12-year-old children, the fantasy subcomponent of trait empathy was directly associated with the enjoyment of sad music, mirroring patterns previously observed in adults (Eerola et al., 2021; Sachs et al., 2015; Vuoskoski et al., 2012). More recently, Carraturo et al. (2022) reported that empathy significantly predicted BMRQ scores in both adults and a small sample of preadolescents (M age = 10.5 years), outperforming objective measures of musical ability. Converging evidence at the neural level further indicates that in preadolescents individual differences in BMRQ-measured musical reward are reflected in the engagement during music listening of reward-related brain networks that substantially overlap with neural systems supporting affective and socio-emotional processing (Fasano et al., 2023).

Together, these studies raise the possibility that socio-emotional traits may already shape musical reward sensitivity in late childhood and preadolescence. However, it remains unclear whether this association is already present at earlier developmental stages, before music engagement becomes more increasingly intertwined with identity formation, peer affiliation, and emotional self-regulation, and autonomous music use during adolescence.

Investigating middle childhood is therefore theoretically important because it allows examination of musical reward sensitivity at a developmental stage in which socio-emotional capacities are rapidly developing, while many of the identity-related and self-directed functions of music that characterize adolescence are still comparatively less prominent. In particular, this developmental period is characterized by continued refinement in affective empathy, self-other distinction, and emotional understanding (Pons et al., 2004; Decety and Cowell, 2014; Eisenberg et al., 2015). By contrast, adolescence has consistently been described as a stage in which music assumes increasingly important functions for identity expression, peer affiliation, emotional self-regulation, and social positioning (Saarikallio and Erkkila, 2007; McPherson and Williamon, 2016; Miranda and Claes, 2009; North et al., 2000; Roberts et al., 2009). Examining the empathy-musical reward association before these functions become more central may therefore help clarify whether musical reward sensitivity is already linked to socio-emotional dispositions independently of later emerging identity-related and autonomy-driven uses of music.

Importantly, validated self-report measures of empathy can be reliably administered in this age range. The Empathy Questionnaire for Children and Adolescents (EmQue-CA), originally validated for children aged 10–15 (Overgaauw et al., 2017), has shown good psychometric properties in younger samples aged 7–12 (Lazdauskas and Nasvytiene, 2021; Liang et al., 2022; Lin et al., 2021). This makes middle childhood a suitable developmental stage for investigating associations between self-perceived empathy and musical reward sensitivity.

Addressing these questions, however, requires age-appropriate measurement of musical reward itself. While the BMRQ is well established in adults, recent work suggests that its factorial structure may not be invariant across development. Kragness et al. (2024), using a parent-report adaptation in preschoolers, reported a factor structure that diverged from the adult model and discussed limitations in the developmental applicability of some adult-oriented item content. At the opposite end of childhood development, Lippolis et al. (2025) identified a substantially different factor structure in adolescents compared to the original adult BMRQ model. To date, no study has formally examined the psychometric structure and validity of the BMRQ in middle childhood, leaving a critical gap between preschool and adolescent research.

Establishing a reliable and age-appropriate measurement model in this age range is a necessary prerequisite for interpreting any associations between musical reward and socio-emotional traits. In other words, evidence that empathy relates to musical reward in children is only meaningful to the extent that the BMRQ captures stable individual differences in music-related reward sensitivity at this developmental stage.

In addition to empathy, other musical characteristics have been considered as potential correlates of musical reward. Musical engagement, reflecting the frequency and diversity of everyday interactions with music, has been associated with stronger reward responses in adults and adolescents (Chapin et al., 2010; Gold et al., 2013; Lippolis et al., 2025). Musical ability represents a related but theoretically distinct construct. While some studies suggest that musical expertise may be associated with differences in emotion recognition and in the relationship between felt emotion and liking (Brattico et al., 2016; Castro and Lima, 2014), developmental findings using the BMRQ have been inconsistent. Carraturo et al. (2022) found no association between musical ability and musical reward in children, and Kragness et al. (2024) similarly reported null associations in preschoolers. These mixed results highlight the importance of considering musical ability as a complementary, exploratory factor when examining individual differences in musical reward during childhood.

The present study addresses these gaps by pursuing two complementary aims in children aged 7–9 years, a developmental stage in which socio-emotional capacities are rapidly developing while music-related identity processes are still comparatively less central than in adolescence.

First, we examine the psychometric structure of a child-adapted Italian version of the BMRQ, including its applicability across gender, to assess whether musical reward sensitivity can be reliably assessed in middle childhood. Given the original theoretical formulation of the BMRQ (Mas-Herrero et al., 2013), we expected the core multidimensional structure of musical reward to be observable in middle childhood as well. At the same time, based on previous developmental adaptations showing age-related modifications in the factorial organization of musical reward (Kragness et al., 2024; Lippolis et al., 2025), we considered the possibility that some dimensions, particularly those related to autonomous music seeking, might show weaker psychometric performance in this age range.

Second, we investigate the association between musical reward sensitivity and empathy. Based on previous findings linking empathy to musical reward in adults and preadolescents, we expected musical reward sensitivity to be positively associated with empathy, particularly its affective component. Associations between musical reward, everyday musical engagement, and objective musical ability were examined as secondary, exploratory analyses, given the still limited developmental evidence on these relationships and in order to contextualize the empathy-reward relationship within a broader developmental framework.

## Materials and methods

### Participants

A total of 651 children were recruited. Only children providing sufficient responses to compute the BMRQ total score were included in the main analyses. The final data sample consisted of 621 children (49.1% girls), aged 7–9 years (M = 8.73, SD = 0.91). This study was carried out as part of a larger multi-year action-research program on music education and child development. Details of the sample characteristics are presented in Table 1.

**Table 1:** Demographic characteristics of the sample by gender, school grade, and age.

Gender	Grade	*N* (%)	Age, Mean ±SD
Girls	2nd grade	162 (26.1%)	8.24 ± 0.62
	3rd grade	143 (23.0%)	9.26 ± 1.09
Boys	2nd grade	167 (26.9%)	8.27 ± 0.59
	3rd grade	149 (24.0%)	9.35 ± 0.61
Total		621 (100%)	8.73 ± 0.91

### Procedure

The study protocol was approved by the University of Aosta Ethics Committee (prot. 0019336, 30/11/2022). Informed consent was obtained in writing from the parents or caregivers on behalf of the child participants. Both the parents/caregivers and the children were informed that they could withdraw from participation at any time.

Children were tested in the local school's computer lab and completed the assessments individually and privately. Each child had access to a personal computer or tablet and was provided with individual earphones to ensure audio clarity and minimize distractions. Sessions were administered by trained personnel under the supervision of a licensed psychologist. Administrators read aloud the instructions and questionnaire items to facilitate comprehension and support children's reading abilities, while responses were completed individually by each child on the device using the mouse. Each child completed the tasks in approximately one and a half hours, with data collection for the full sample completed over a 3-week period.

### Materials

**Barcelona Music Reward Questionnaire—Middle Childhood version (BMRQ-MC)**. A 19-item Italian child-adapted version of the Barcelona Music Reward Questionnaire (BMRQ; Mas-Herrero et al., 2013) was used to assess the degree of musical reward sensitivity. Items 2 and 5 were reverse-scored. The BMRQ includes five subscales: Music Seeking, Emotion Evocation, Mood Regulation, Sensory-Motor, and Social Reward. Children responded using a five-point Likert scale ranging from 1 (“strongly disagree”) to 5 (“strongly agree”). The Italian child-adapted version was developed using a forward–backward translation procedure. First, all original BMRQ items were translated from English into Italian by two bilingual researchers with expertise in developmental psychology and music cognition. A different bilingual translator, blind to the original wording, independently back-translated the items into English. Discrepancies between the original and back-translated versions were discussed until conceptual equivalence was achieved. Although all items were translated and linguistically simplified for age-appropriate comprehension while remaining as close as possible to the original adult scale, one item required more substantial developmental rewording beyond direct translation to ensure age-appropriate comprehension. In particular, the original item “I spend quite a bit of money on music and related items” was adapted to “My parents and I spend a lot of money on music and musical instruments” to reflect the reality that children typically do not purchase music independently. Moreover, prior to data collection, a pilot study with 10 children aged 7–9 was conducted to assess comprehension of the translated 20-item version. One item (“I inform myself about music I like”) was not understood by most children. Notably, Kragness et al. (2024) reworded the same item for use with preschoolers, but even the adapted version was ultimately excluded from their final model due to poor psychometric fit. Based on both the lack of comprehension in our pilot sample and prior evidence suggesting that certain aspects of musical seeking may be developmentally inappropriate for younger populations, this item was excluded before the main data collection, resulting in a 19-item child-adapted version used in the present study. The full list of BMRQ-MC items, corresponding subscales, and item numbering used in the factor loading figure is reported in Supplementary Table S1 in the Supplementary material.

**EmQue-CA** (Empathy Questionnaire for Children and Adolescents; Liang et al., 2022). This 14-item self-report instrument using a three-point Likert scale (from “not true” to “often true”) was used to measure empathy across three subscales: Affective Empathy (6 items), Cognitive Empathy (3 items), and Prosocial Motivation (5 items). The EmQue-CA showed good internal consistency in the present sample (Cronbach's α = 0.77).

**CCM** (Concurrent Musical Activities questionnaire; Müllensiefen et al., 2015). A brief Music Activity questionnaire adapted from the CCM developed for school-aged students (Müllensiefen et al., 2015) was used to index instrument playing/lessons, practice frequency and duration, and ensemble participation. The questionnaire consisted of eight yes/no items assessing different types of ongoing musical activities, such as taking individual or group music lessons, playing music during special occasions, and participating in music activities at school (Müllensiefen et al., 2015). The items were combined into a single activity score (higher = greater involvement). The CCM was designed as a brief tool to capture the level of ongoing musical activities among students (Müllensiefen et al., 2015).

**MBEMA** (Montreal Battery for Evaluation of Musical Abilities; Peretz et al., 2013). Musical perceptual abilities were measured with two subtests from the abbreviated child-friendly version of the MBEMA (Peretz et al., 2013). In both subtests, children heard pairs of short musical sequences and judged whether they were the same or different. Each subtests included 20 trials, comprising 10 trials with identical comparison melodies and 10 trials with different comparison melodies. In the melodic discrimination (scale) test, trials in which the melodies differed contained a single scale-violating out-of-key note while preserving melodic contour. In the rhythmic discrimination test, trials in which the melodies differed were created through changes in the durations of two adjacent notes, altering the rhythmic grouping of notes while preserving melodic content, original meter, and number of notes. Each subtest included unfamiliar tonal melodies (5–9 notes; ~3–4 s) presented in varied timbres. Scores were calculated as the percentage of correct judgments for each subtest.

### Statistical analysis

First, a Confirmatory Factor Analysis (CFA) was conducted on the 19 items of the child-adapted Barcelona Music Reward Questionnaire (BMRQ-MC) using Jamovi software (version 2.2.5) and the “lavaan” (Rosseel, 2012), “semTools” (Jorgensen et al., 2022) package for the multigroup CFA. For the correlational analyses and independent *t*-tests SPSS (version 27.0) was used. Sample sizes varied slightly across analyses due to missing data on specific measures.

The following indexes were used to assess the fit of the CFA models: (1) chi-square (χ^2^); (2) Comparative Fit Index (CFI; acceptable fit ≥0.90); (3) Tucker-Lewis Index (TLI; acceptable fit ≥0.90); and (4) Root Mean Square Error of Approximation (RMSEA; acceptable fit ≤ 0.08). Internal consistency of the scale was computed using McDonald's omega and Cronbach's alpha coefficients.

Second, the model was tested separately on two groups (i.e., girls, *N* = 305, and boys, *N* = 316) to establish configural invariance (van de Schoot et al., 2012). In addition, to test whether the BMRQ-MC elicited similar responses across the two groups we conducted a multigroup CFA, which is a statistical method that examines the observed differences in a measurement model, known as measurement invariance (Byrne et al., 1989). The initial phase of assessing measurement invariance involves configural invariance, wherein a model is constructed to determine if the items load onto the same underlying factor. The subsequent step is metric invariance, where factor loadings are constrained to be equivalent across groups. Achieving metric invariance allows for the comparison of correlation coefficients between groups. The final step is scalar invariance, which imposes uniformity on item intercepts across groups. Metric and scalar invariances are attained if: (a) the changes in CFI and RMSEA between models are negligible and however below 0.01 and 0.015, respectively (Cheung and Rensvold, 2002); specifically, 0.010 was recommended for testing metric invariance with *N* < 300, with all factor loadings in one group established to be higher than in the other, and unequal group sample sizes, while a cutoff of 0.015 was recommended for evaluating metric invariance with *N* > 300 (Beribisky and Hancock, 2024); and, (b) the multi-group model fit indexes indicate a good fit (Beaujean et al., 2012).

Independent-samples *t*-tests were conducted in order to test mean differences in BMRQ-MC scores between boys and girls. In addition, complementary exploratory independent-samples *t*-tests were conducted to examine potential gender differences in CCM, and MBEMA scores.

Pearson's correlation coefficients were computed to examine the associations between BMRQ-MC scores and empathy (EmQue CA) to establish evidence of convergent validity. Additional exploratory correlations were conducted between BMRQ-MC scores and musical engagement (CCM) as well as objective musical abilities (MBEMA).

Finally, a hierarchical multiple regression analysis was performed to assess the unique contribution of empathy, musical engagement, and musical abilities to musical reward sensitivity. The total BMRQ-MC score was entered as the dependent variable, with predictors entered in successive steps. Assumptions of linear regression were evaluated following standard diagnostic procedures. Multicollinearity was assessed using variance inflation factors, and independence of residuals was examined using the Durbin-Watson statistic. Linearity and homoscedasticity were inspected through scatterplots of standardized residuals against predicted values, and normality was evaluated using histograms and Q-Q plots of standardized residuals. All assumptions were satisfactorily met.

## Results

**Figure 1:**
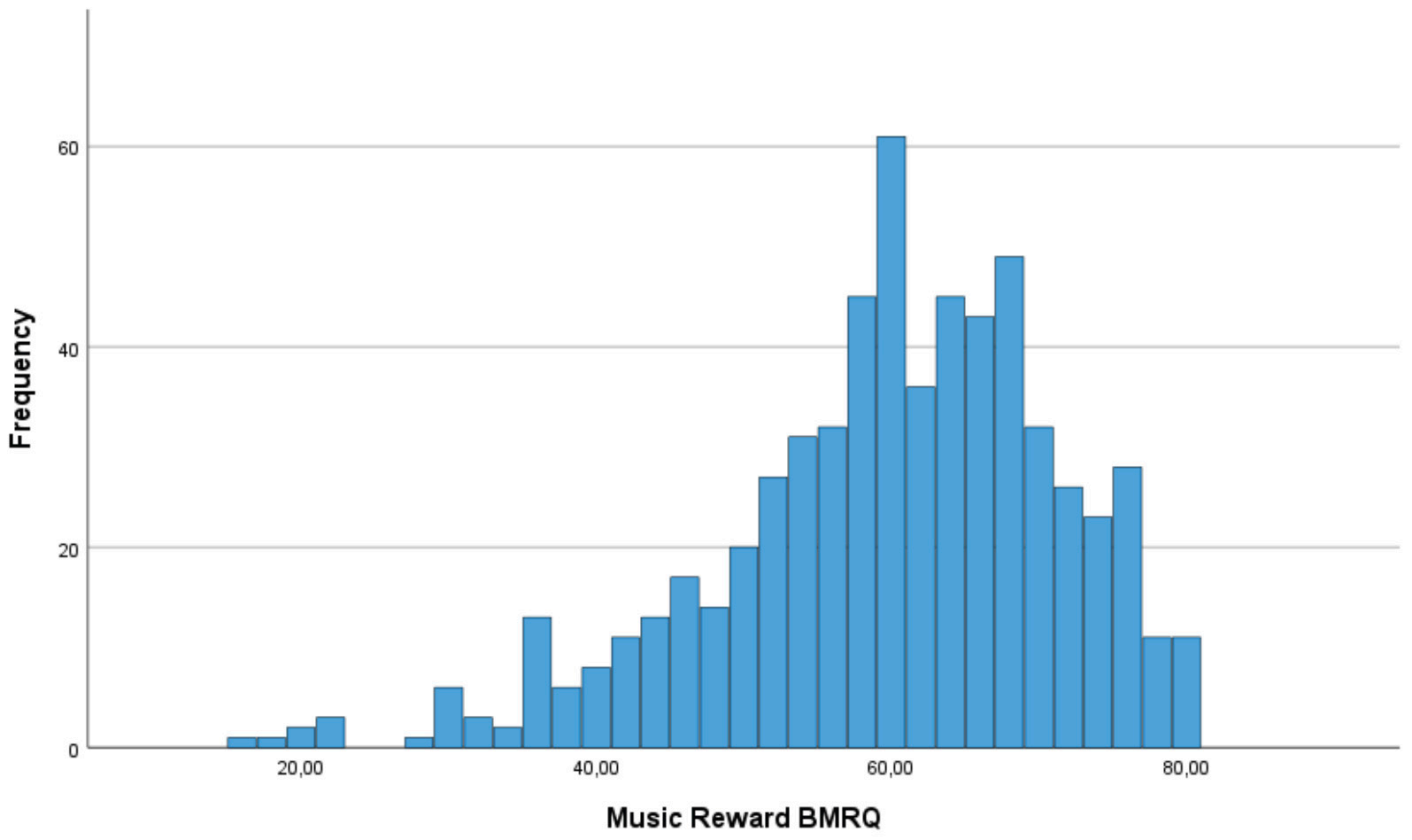
Distribution of total scores on the reduced four-factor BMRQ-MC in children aged 7–9 years.

Descriptive inspection of the reduced four-factor BMRQ-MC global scores indicated a broad distribution without evidence of ceiling effects, suggesting that the scale captured substantial interindividual variability in musical reward sensitivity in this age group (Figure 1).

### Confirmatory factor analysis and measurement invariance

A confirmatory factor analysis (CFA) was conducted to test the latent structure of the child-adapted BMRQ-MC in children, following the original five-factor theoretical model proposed by Mas-Herrero et al. (2013). The model included the following factors: Social Reward, Sensory Motor, Mood Regulation, Musical Seeking, and Emotional Evocation.

The CFA performed on the considered 19 BMRQ-MC items for the global model showed an adequate fit to the data: χ^2^(142) = 369, *p* < 0.001, CFI = 0.905, TLI = 0.885, SRMR = 0.045, RMSEA = 0.051 [0.044–0.057]. Although the CFI was slightly below the conventional cutoff of 0.95 (Hu and Bentler, 1999), the TLI and RMSEA values fell within acceptable ranges (Hu and Bentler, 1999), indicating that the overall fit of five-factor model was satisfactory.

All items showed significant loadings on their respective factors (all *p*s < 0.001), with standardized values generally ranging from 0.44 to 0.71 for Social reward, −0.71 to −0.33 for Sensory motor, 0.52–0.68 for Mood regulation, −0.20 to 0.56 for Musical seeking, and 0.47–0.56 for Emotional evocation. These loadings indicate that the observed items converged satisfactorily on their intended latent constructs, supporting the convergent validity of the measurement model. However, two of the three items from the subscale Musical seeking (e.g., Music_reward_2 “In my free time I almost never listen to music” and Music_reward_16 “My parents and I spend a lot of money on music and musical instruments”) showed relatively low loadings. Residual analysis showed some local discrepancies, but no *post-hoc* modifications were deemed necessary to significantly improve the model. Although modification indexes suggested potential cross-loadings (e.g., Music_reward_16 on other factors), such interventions were not applied in order to preserve the theoretical consistency of the model.

The internal consistency of the total scale's scores was excellent, with Cronbach alpha = 0.84 and McDonald's omega = 0.84. At the subscale level, Cronbach's alpha and McDonald's omega showed moderate-good ranging values for four of the five factors (Social Reward α = 0.64, ω = 0.65; Sensory Motor α = 0.61, ω = 0.63; Mood Regulation α = 0.71, ω = 0.72; Emotional Evocation α = 0.62, ω = 0.63), exceeding the minimum acceptable threshold of 0.60 for composite reliability (Hair et al., 2019). In contrast, the Musical Seeking subscale yielded unsatisfactory internal consistency (α = 0.16, ω = 0.41).

Given the unsatisfactory reliability of the Musical Seeking subscale, together with the previously noted weak factor loadings for two of its three items in the subscale (Music_reward_2 and Music_reward_16), the Musical Seeking factor was excluded from subsequent analyses. Importantly, this subscale already comprised one item fewer than in the original BMRQ, as one item had been removed during piloting due to limited comprehensibility in this age group. The remaining items still refer to behaviors such as autonomous music discovery or spending money on music, which may be less representative or meaningful at this age. Taken together with prior considerations regarding the developmental appropriateness of Musical Seeking content (Kragness et al., 2024), these findings suggest that this dimension, as operationalized in the original questionnaire for adults, may be less suitable for children in middle childhood.

**Figure 2:**
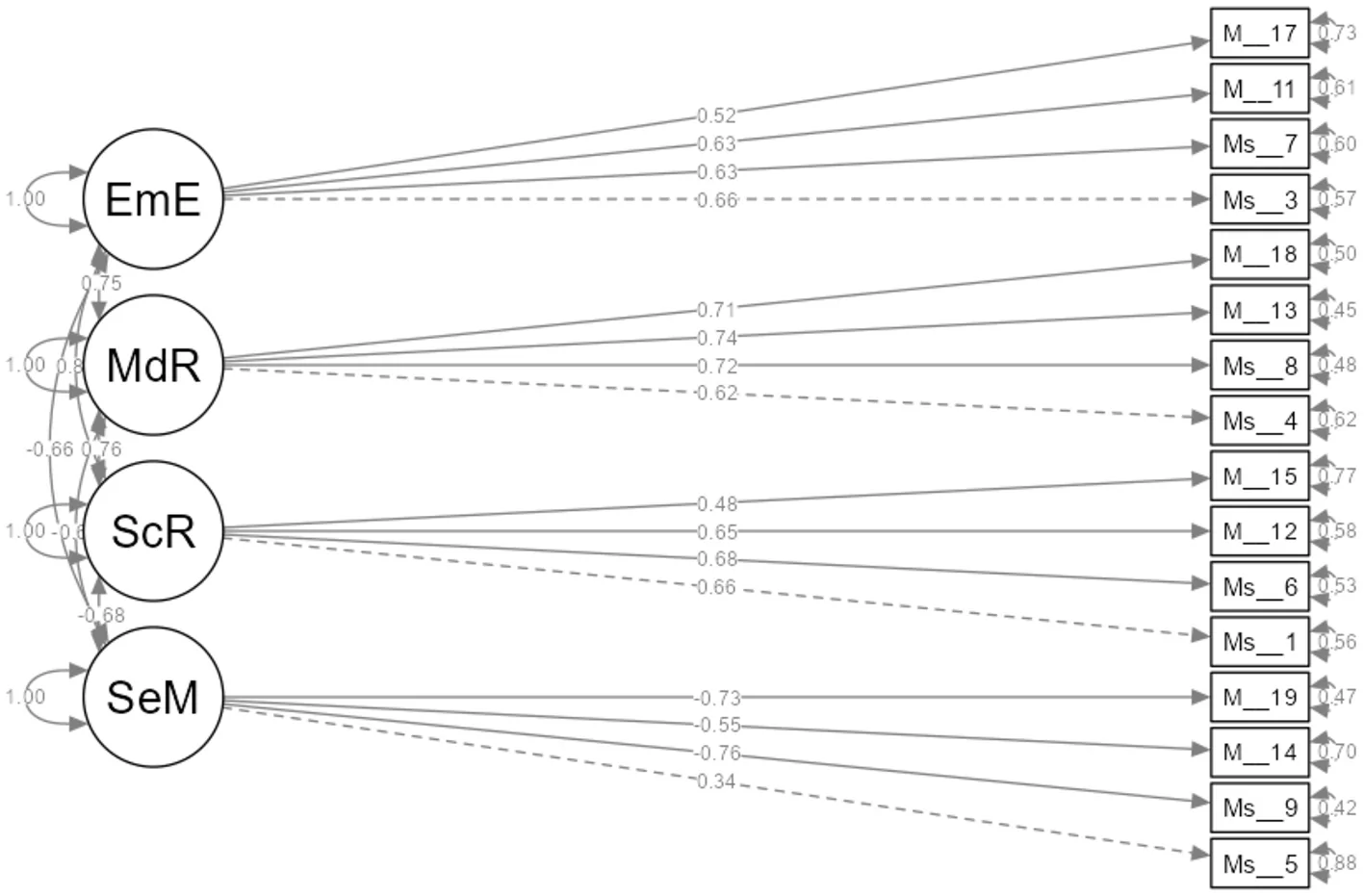
CFA loadings of the four-factor solution. Beta coefficients and residuals are reported. EmE, emotion evocation; MdR, mood regulation; ScR, social reward; SeM, sensory motor.

Therefore, a second CFA was conducted on a reduced four-factor model excluding the Musical Seeking factor. All fit indexes improved considerably, with a significant difference at the chi-square difference test, and a substantial improvement in CFI, TLI, and RMSEA. The four factors solution with item loadings is reported in Figure 2.

To examine whether the four-factor model was invariant across gender, the CFA was run separately for girls and boys. The model demonstrated excellent fit in both groups, with slightly better fit indices in girls [girls: χ^2^(98) = 100, *p* ≤ 0.001, CFI = 0.999, TLI = 0.989, RMSEA = 0.009; boys: χ^2^(98) = 172, *p* < 0.001, CFI = 0.981, TLI = 0.987, RMSEA = 0.048], suggesting that the scale could constitute a good measure for both groups.

Regarding model invariance, the fit indexes of the unconstrained multi-group model [χ^2^(196) = 272, *p* < 0.001, CFI = 0.990, TLI = 0.988, RMSEA = 0.035] demonstrated the configural invariance of the model across groups, suggesting that the factor structure is similar across girls and boys. In the subsequent metric model, all item loadings were constrained to equality and differences in fit indexes did not reveal globally a significant reduction in model fit [Δχ^2^(12) = 42, *p* = ns, ΔCFI = 0.004, ΔTLI = 0.004, ΔRMSEA = 0.006], suggesting that the meaning of the construct assessed by BMRQ-MC is similar across both girls and boys. Finally, all the item thresholds were constrained across groups to test for scalar invariance. Results showed that the fit of the scalar model is not significantly different than the metric model [Δχ^2^(4) = 0, *p* = ns, ΔCFI = 0.003, ΔTLI = 0.003, ΔRMSEA = 0.004], therefore not significantly worse than the previous one thus supporting invariance.

Overall, these results support configural, metric, and scalar invariance of the four-factor BMRQ-MC across gender. All subsequent analyses were therefore conducted using the four-factor structure, and the BMRQ-MC global score was computed accordingly. Model fit indices for the original five-factor model and the reduced four-factor solution, as well as measurement invariance results across gender, are reported in Table 2.

**Table 2:** Fit indexes for the five-factors and four-factors models and for measurement invariance testing of the BMRQ-MC across girls and boys.

Model	*N*	*Δχ*^2^(df)	*Δχ*^2^(df)	CFI	ΔCFI	TLI	ΔTLI	RMSEA (CI)	ΔRMSEA
5 Factors	621	369 (142)		0.905		0.885		0.0507 (0.44–0.57)	
4 Factors	621	240 (98)	129 (44)^***^	0.931	0.026	0.916	0.031	0.0483 (0.041–0.046)	0.0024
Girls	305	100 (98)		0.999		0.999		0.009 (0.00–0.032)	
Boys	316	172 (98)		0.981		0.977		0.049 (0.037–0.061)	
Configural	621	272 (196)		0.990		0.988		0.035 (0.025–0.045)	
Metric	621	314 (208)	42 (12)	0.986	0.004	0.984	0.004	0.041 (0.031–0.049)	0.006
Scalar	621	272 (192)	0 (4)	0.989	0.003	0.987	0.003	0.037 (0.26–0.046)	0.004

^***^p < 0.001.

Following the measurement invariance analyses, exploratory independent-samples *t*-test were conducted to examine mean-level gender differences in BMRQ-MC scores. Girls scored significantly higher than boys on the BMRQ-MC global score, *t*(619) = 6.53, *p* < 0.001 (girls: M = 3.89, SD = 0.64; *n* = 305; boys: M = 3.53, SD = 0.77; *n* = 316). The same pattern emerged across all four retained BMRQ-MC subscales. Girls reported higher scores than boys on Social Reward, Sensory-Motor, Mood Regulation, and Emotional Evocation (all *p*s ≤ 0.001). Complementary exploratory analyses showed no significant gender differences for CCM or MBEMA scores (all *p*s > 0.05).

### Correlational analyses

Pearsons correlations were computed to examine associations between musical reward, empathy, musical engagement, and musical ability.

To assess convergent validity and examine theoretically motivated associations between musical reward and socio-emotional traits, correlations were computed between BMRQ-MC scores and empathy as measured by EmQue-CA. The global BMRQ-MC score was strongly and positively correlated with overall empathy, *r* = 0.490, *p* < 0.001, thus providing evidence for convergent validity. In line with Westen and Rosenthal (2003), a moderate correlation sustains the association between the two constructs without being overlapped. Moreover, at the subscale level, as shown in Table 3, all the dimensions of our scale were found to have a significant positive association with empathy subscales (affective, cognitive, and prosocial empathy), with affective empathy showing the strongest correlation with emotion evocation subscale of the BMRQ-MC, *r* = 0.468, *p* < 0.001.

**Table 3:** Pearson correlations between BMRQ-MC subscales and empathy dimensions (EmQue-CA).

Variables		Affective empathy	Cognitive empathy	Prosocial empathy
Emotion evocation	Pearson correlation	0.468^*^	0.176^*^	0.248^*^
	Sig. (two tailed)	< 0.001	< 0.001	< 0.001
Social reward	Pearson correlation	0.398^*^	0.252^*^	0.308^*^
	Sig. (two tailed)	< 0.001	< 0.001	< 0.001
Mood regulation	Pearson correlation	0.348^*^	0.176^*^	0.301^*^
	Sig. (two tailed)	< 0.001	< 0.001	< 0.001
Sensory motor	Pearson correlation	0.243^*^	0.135^*^	0.208^*^
	Sig. (two tailed)	< 0.001	< 0.001	< 0.001

^*^p < 0.05.

With respect to musical engagement, measured through the CCM, a small but significant positive correlation was found with the BMRQ-MC global score (Table 4). At the subscale level, musical engagement was positively associated with Social Reward (*r* = 0.175, *p* < 0.001), while correlations with Mood Regulation, Emotional Evocation, and Sensory-Motor were non-significant.

**Table 4:** Pearson correlations between BMRQ-MC, EmQue-CA, CCM, MBEMA.

Variables		BMRQ	EmQue-CA	CCM	MBEMA rhythm	MBEMA melody
BMRQ-MC	Pearson correlation	1			–	–
	Sig. (two tailed)				–	–
EmQue-CA	Pearson correlation	0.490^*^	1		–	–
	Sig. (two tailed)	< 0.001			–	–
CCM	Pearson correlation	0.111^*^	0.005	1	–	–
	Sig. (two tailed)	0.009	0.904		–	–
MBEMA rhythm	Pearson correlation	ns	ns	ns	1	–
	Sig. (two tailed)				–	–
MBEMA melody	Pearson correlation	ns	ns	ns	0.794^*^	1
	Sig. (two tailed)				< 0.001	–

^*^Correlation is significant at the 0.01 level (2-tailed).

Regarding musical ability, assessed through the MBEMA melody and rhythm discrimination scores, no significant association emerged with the BMRQ-MC global score (melody: *r* = −0.014, *p* = 0.722; rhythm: *r* = −0.031, *p* = 0.439). Correlations between MBEMA scores and BMRQ-MC subscales were generally small and inconsistent. Specifically, weak negative correlations were observed between Emotional Evocation and both melody (*r* = −0.082, *p* = 0.040) and rhythm discrimination (*r* = −0.120, *p* = 0.003). No other significant associations were found.

Finally, exploratory correlations with age were examined. No significant associations emerged for the global BMRQ-MC score or for any subscale except Sensory-Motor, which showed a small negative correlation with age (*r* = −0.135). Given the restricted age range of the sample and the small magnitude of the observed association, age was not included in subsequent analyses.

### Regression analysis

A hierarchical multiple regression analysis was conducted to examine the contribution of empathy, musical engagement, and musical abilities to musical reward sensitivity (see Table 5). The BMRQ-MC global score (reduced four-factor score) was entered as the dependent variable. To account for the observed gender differences in musical reward sensitivity, gender was entered as a control variable in the first step of the model, followed by empathy as the primary theory-driven predictor, and everyday musical engagement and objective musical ability as secondary exploratory predictors.

**Table 5:** Hierarchical regression analysis considering music reward as dependent variable.

Model	Predictors	*R*	*R* ^2^	Adjusted *R*^2^	SE of estimate	Δ*R*^2^	*F* change	df1	df2	Sig. Δ*F*	Durbin-Watson
1	Gender	0.267	0.071	0.070	0.704	0.071	42.36	1	551	< 0.001	
2	Gender, EmQue-CA	0.526	0.277	0.274	0.622	0.205	156.26	1	550	< 0.001	
3	Gender, EmQue-CA, CCM	0.537	0.288	0.284	0.618	0.011	8.69	1	549	0.003	
4	Gender, EmQue-CA, CCM, MBEMA Melody, MBEMA Rhythm	0.539	0.291	0.284	0.618	0.003	1.07	2	547	0.346	1.86

In the first step, gender was entered into the model. This model was statistically significant, *F*_(1, 551)_ = 42.36, *p* < 0.001, explaining 7.1% of the variance in musical reward (*R* = 0.267, *R*^2^ = 0.071, adjusted *R*^2^ = 0.070). Gender was a significant predictor of musical reward sensitivity (β = −0.267, *p* < 0.001).

In the second step, total empathy was added to the model. Empathy explained an additional 20.5% of the variance, Δ*R*^2^ = 0.205, *F* change_(1, 550)_ = 156.26, *p* < 0.001, resulting in a total explained variance of 27.7% (*R* = 0.526, *R*^2^ = 0.277, adjusted *R*^2^ = 0.274). In this model, both gender (β = −0.244, *p* < 0.001) and empathy (β = 0.454, *p* < 0.001) were significant predictors, with empathy showing the strongest association with BMRQ-MC scores.

In the third step, everyday musical engagement (CCM) was added to the model. The inclusion of CCM led to a small but statistically significant increase in explained variance (Δ*R*^2^ = 0.011), resulting in a total explained variance of 28.8% (*R* = 0.537, *R*^2^ = 0.288, adjusted *R*^2^ = 0.284), *F* change_(1, 549)_ = 8.69, *p* = 0.003. Gender (β = −0.241, *p* < 0.001), empathy (β = 0.453, *p* < 0.001) and musical engagement (β = 0.106, *p* = 0.003) were all significant predictors of musical reward.

In the fourth step, musical abilities were entered into the model using MBEMA melody and rhythm scores. The addition of these variables did not result in a significant increase in explained variance (Δ*R*^2^ = 0.003), *F* change_(2, 547)_ = 1.07, *p* = 0.346. The final model explained 29.1% of the variance in musical reward sensitivity (*R* = 0.539, *R*^2^ = 0.291, adjusted *R*^2^ = 0.284). Empathy remained the strongest predictor of musical reward (β = 0.454, *p* < 0.001), followed by gender (β = −0.243, *p* < 0.001) and musical engagement (β = 0.108, *p* = 0.003). Neither MBEMA melody (β = −0.039, *p* = 0.492) nor MBEMA rhythm scores (β = −0.077, *p* = 0.179) were significantly associated with BMRQ-MC scores.

Diagnostic checks indicated no violations of regression assumptions. Residuals were approximately normally distributed, showed no evidence of autocorrelation (Durbin-Watson = 1.86), and variance inflation factors indicated no multicollinearity issues (all VIFs < 3).

## Discussion

The present study investigated musical reward sensitivity in middle childhood by addressing two complementary aims: the psychometric validation of the BMRQ-MC in children aged 7–9 years, and the examination of its associations with empathy, everyday musical engagement, and musical ability. By doing so, the study extends the application of the BMRQ to a developmental window that has so far received limited attention and provides new insights into the socio-emotional foundations of musical reward at an age preceding adolescence-related changes in identity formation, peer orientation, and self-directed music use. More specifically, the findings suggest that musical reward in middle childhood is already closely linked to socio-emotional dispositions, while dimensions requiring greater autonomy and self-directed engagement, such as Musical Seeking, may become more central only later in development.

### Psychometric structure of the BMRQ-MC in middle childhood

The confirmatory factor analyses provided partial support for the original five-factor structure of the BMRQ proposed by Mas-Herrero et al. (2013). While the overall model showed an acceptable fit to the data, the Musical Seeking subscale exhibited weak factor loadings and very poor internal consistency, leading to its exclusion from subsequent analyses. In contrast, the remaining four dimensions, Emotional Evocation, Mood Regulation, Social Reward, and Sensory-Motor, showed satisfactory psychometric properties and a coherent latent structure.

This pattern is consistent with prior developmental adaptations of the BMRQ. Kragness et al. (2024), using a parent-report version in preschool children, reported a factor structure that diverged from the adult model, whereas Lippolis et al. (2025) identified a substantially different factor solution in adolescents. Together, these findings indicate that musical reward sensitivity is not structurally invariant across development and that its components may emerge, differentiate, or stabilize at different developmental stages.

At the same time, developmental differences may not only reflect changes in the organization of a stable construct, but also age-specific ways through which musical reward is experienced and expressed. As suggested by Kragness et al. (2024), musical reward in childhood may be more strongly embedded in socially shared, playful, and externally structured musical activities than in the autonomous and self-directed music engagement more characteristic of adolescence and adulthood. In this sense, some dimensions of musical reward in children may not be fully captured by questionnaires originally developed for adult populations.

This interpretation may be particularly relevant for the Musical Seeking dimension, whose items presuppose levels of autonomy and self-directed music engagement that are less typical of middle childhood. Several items in this subscale refer to autonomous music exploration, deliberate choice seeking, or financial investment in music, all behaviors that presuppose levels of agency, independence, and access that are not typical of children aged 7–9 years (Kragness et al., 2024). More generally, research on survey methodology in childhood has repeatedly shown that self-report items requiring abstract reasoning, autonomy, or future-oriented decision-making are particularly problematic in younger respondents and often show reduced reliability and validity (Borgers et al., 2000; DeVellis and Thorpe, 2022). Consistent with this interpretation, Kragness et al. (2024) note that musical seeking behaviors show fewer clear parallels in the developmental literature, as children rarely have the opportunity to purchase music or to independently seek musical information in ways assumed by adult questionnaires.

This developmental account is further supported by research on music use showing that self-directed music exploration increasingly serves identity expression and self-regulation primarily during adolescence, alongside growth in autonomy and self-concept differentiation (McPherson and Williamon, 2016; Saarikallio, 2019). Accordingly, Musical Seeking may represent a dimension of musical reward that becomes more meaningful later in development (Lippolis et al., 2025). Nevertheless, this does not necessarily imply an absence of musical autonomy in childhood. Rather, children's autonomous engagement with music may emerge through developmentally specific behaviors, such as choosing musical toys, requesting particular songs, or interacting with music through digital devices and family contexts (Lamont, 2008), which may not be fully captured by adult-oriented Musical Seeking items. After removal of this subscale, the four-factor model showed improved fit, supporting the use of a reduced BMRQ-MC structure for group comparisons and correlational analyses in middle childhood.

The reduced four-factor model also showed measurement invariance across gender, indicating that the retained structure operated similarly for girls and boys. This finding supports the use of the BMRQ-MC for group comparisons in this age range. Within this invariant measurement structure, girls reported higher musical reward sensitivity than boys across the global score and the retained subscales. This pattern is consistent with previous BMRQ studies reporting higher sensitivity to musical reward among girls in preadolescence and adolescence, as well as with adult findings showing stronger self-reported emotional responses to music among women (Lippolis et al., 2023, 2025; Mas-Herrero et al., 2013; Wang et al., 2023). Importantly, no corresponding gender differences emerged for everyday musical engagement or objective musical ability, suggesting that the observed differences in BMRQ-MC scores are unlikely to be explained simply by higher current musical participation or better perceptual musical skills among girls. Taken together, these findings suggest that, in middle childhood, girls may report greater sensitivity to the rewarding aspects of music even when they do not differ from boys in their current musical engagement or objective musical ability.

### Empathy and musical reward: evidence for convergent validity

In line with the primary theoretical hypothesis, musical reward sensitivity showed a robust and positive association with empathy. The correlation between the BMRQ-MC global score and overall empathy was moderate in magnitude, providing clear evidence for convergent validity without indicating construct redundancy. At the subscale level, affective empathy showed the strongest associations, particularly with Emotional Evocation, suggesting that children who are more prone to emotionally resonate with others (e.g., “If a friend is sad, I feel sad as well”) also report stronger emotional responses to music (e.g., “I get emotional listening to certain pieces of music”).

This pattern is theoretically grounded in contemporary models of empathy, which distinguish affective sharing from cognitive perspective taking (Decety and Jackson, 2004). Music engages affective resonance mechanisms such as emotional contagion, which have been proposed as key routes through which expressive musical cues elicit felt emotion and pleasure (Juslin and Västfjäll, 2008). Beyond this descriptive account, converging neural evidence suggests that music-evoked reward involves dynamic interactions between systems supporting affective salience and those supporting reward valuation. In preadolescents, individual differences in BMRQ-measured musical reward sensitivity have been shown to relate to transient engagement of insular and salience-related regions followed by increased recruitment of reward-related networks during music listening (Fasano et al., 2023). Such dynamics are consistent with the notion that affective resonance may act as an entry point through which emotionally salient musical stimuli gain access to reward-related processing, providing a plausible neural bridge between empathy-related dispositions and musical pleasure.

The present findings extend this evidence to younger children and demonstrate that the empathy-musical reward association is already robust by early primary school age. Importantly, this association emerges before music typically assumes the stronger identity-related, peer-oriented, and self-regulatory functions that characterize adolescence. The present results therefore suggest that individual differences in musical reward may initially emerge in close connection with socio-emotional dispositions, before later developmental processes increasingly shape how music is used and experienced. In this sense, the findings support the view that empathy-related affective resonance may represent an early correlate of musical pleasure. At the same time, the present cross-sectional design does not allow conclusions regarding the direction of this association, and it remains possible that empathy and musical reward sensitivity partly reflect shared underlying socio-emotional or neurodevelopmental factors. Importantly, the observed correlation strengths fall within the range typically considered appropriate for convergent validity (Westen and Rosenthal, 2003), reinforcing the interpretation that empathy and musical reward are related but distinct constructs. This supports the construct validity of the BMRQ-MC in middle childhood and situates musical reward sensitivity within a broader socio-emotional framework.

### Musical engagement, musical ability, and individual differences in musical reward sensitivity

Everyday musical engagement showed a small but significant association with musical reward. This relationship was primarily driven by the Social Reward dimension, suggesting that children who engage more frequently with music also report greater pleasure from shared and socially embedded musical experiences. This finding aligns with developmental research showing that music in childhood often functions as a social activity rather than an individual or skill-focused pursuit (Ilari, 2016), as well as with evidence that collective musical activities and music training are associated with higher social music reward in preadolescence (Lippolis et al., 2023). Unlike findings in adolescence, where musical engagement is more strongly tied to emotional self-regulation and identity-related uses of music (Lippolis et al., 2025), the present pattern suggests that in middle childhood everyday engagement with music may still be grounded mainly in its social and relational functions.

Notably, CCM scores were not significantly associated with MBEMA performance, suggesting that, in middle childhood, frequency of musical engagement may not yet closely map onto perceptual musical skills. Consistently, objective musical ability, assessed through objective measures of melody and rhythm discrimination, also showed no meaningful association with musical reward at the global level. Subscale-level correlations were weak, inconsistent, and occasionally negative. This dissociation closely replicates previous findings in children, where musical reward has been shown to be largely independent of perceptual musical skills (Carraturo et al., 2022), as well as in preschoolers, where musical training does not reliably predict reward sensitivity (Kragness et al., 2024). More broadly, this pattern is consistent with evidence from adult populations demonstrating that musical pleasure can be dissociated from perceptual competence. Studies on musical anhedonia have shown that individuals can display intact music perception while experiencing little or no pleasure from music, indicating that reward processing and perceptual abilities rely on partially distinct mechanisms (Mas-Herrero et al., 2014).

The hierarchical regression analyses further clarified this pattern by estimating the relative contributions of empathy, musical engagement, and musical ability within the same model while controlling for gender. Empathy emerged as the strongest predictor of musical reward sensitivity, explaining an additional 20.5% of the variance beyond gender. Musical engagement explained a much smaller but statistically significant additional share of variance (1.1%), whereas musical abilities did not contribute meaningfully to the model. This pattern closely parallels the regression results reported by Carraturo et al. (2022) and is consistent with broader neurobiological models of reward processing, which emphasize the role of affective and motivational systems over sensory precision in the experience of pleasure (Berridge and Kringelbach, 2015; Kringelbach and Berridge, 2017). The present findings are compatible with this framework in childhood, suggesting that individual differences in empathic responsiveness and children's current engagement in musical activities are more informative than perceptual skill for understanding why some children derive greater pleasure from music than others. Importantly, the marked difference in explained variance indicates that, although both empathy and musical engagement were significant predictors, empathy had considerably greater practical relevance for musical reward sensitivity in middle childhood.

Together, these findings suggest that, in childhood, musical reward is primarily grounded in socio-emotional processes rather than in technical or perceptual proficiency. This perspective may also have implications for educational contexts. Although the present study does not directly evaluate educational interventions or longitudinal change, the observed associations suggest that socio-emotional aspects of musical reward may be relevant when designing music-based educational activities. If musical reward in middle childhood is closely linked to socio-emotional dispositions and socially embedded musical experiences, then music activities in schools may support engagement not only through technical skill acquisition, but also through emotionally and interpersonally meaningful forms of participation. This may be particularly relevant for collective and inclusive musical activities, such as classroom singing, ensemble participation, or shared music-making experiences. Consistent with this interpretation, a growing literature has reported promising socio-emotional and motivational outcomes associated with collective music education and orchestral training programs in childhood, including increased social engagement, cooperative behavior, school-related engagement, and higher social music reward (e.g., Fasano et al., 2025; Lippolis et al., 2023; Ilari et al., 2018; Kirschner and Tomasello, 2010). Musical enjoyment may therefore precede, rather than result from, the development of musical skills and may play a motivational role in later musical learning and engagement (Fasano et al., 2019, 2022; Dai et al., 2025; Sloboda, 2000). Future longitudinal and intervention studies will be necessary to determine whether strengthening socio-emotional engagement with music can promote later musical learning and broader educational outcomes.

### Limitations and future directions

Several considerations should be taken into account when interpreting the present findings.

First, the cross-sectional design precludes causal inferences regarding the directionality of the observed associations. Future longitudinal research will be important to clarify how empathy and musical reward sensitivity influence one another across development. Longitudinal studies spanning middle childhood and adolescence will be particularly important to examine how the socio-emotional and identity-related functions of music progressively evolve and interact across development.

Second, although the reduced four-factor BMRQ-MC structure showed good psychometric properties in this sample, replication in independent cohorts will help establish its broader generalizability. In this context, Emotional Evocation showed moderate rather than high reliability, although item loadings remained significant and theoretically coherent. Future studies may further refine the measurement of specific dimensions of musical reward in middle childhood.

In addition, the present findings suggest that some components of musical reward, particularly those related to autonomous music seeking, may be developmentally constrained in middle childhood. Future studies spanning wider age ranges are needed to determine when these aspects of musical reward become psychometrically and behaviorally meaningful.

Finally, as with many studies in developmental psychology, reliance on self-report measures may have contributed to shared method variance, potentially inflating associations between constructs assessed within the same session (Podsakoff et al., 2003). At the same time, the pattern of findings was theoretically differentiated rather than uniformly strong across measures, with empathy showing robust associations with musical reward, whereas objective musical ability showed no meaningful relationship. This reduces the likelihood that the observed results are solely attributable to generalized response tendencies. Future studies integrating behavioral, observational, and neurophysiological measures would further strengthen the investigation of the relationship between musical reward sensitivity and socio-emotional functioning.

## Conclusions

In conclusion, the present study provides the first psychometric validation of the BMRQ-MC in middle childhood and demonstrates that musical reward sensitivity can be reliably assessed from the age of seven using a reduced four-factor structure. The findings indicate that, at this stage of development, musical reward is strongly associated with empathy, modestly related to everyday musical engagement, and largely independent of objective musical ability.

Importantly, this age range represents a developmental period preceding adolescence-related changes in identity formation, peer orientation, and autonomous music use. The results therefore suggest that, in middle childhood, musical reward sensitivity is more closely tied to socio-emotional dispositions than to self-directed music behaviors or perceptual musical skills. This highlights empathy as a potentially important correlate of early musical pleasure and supports the view that affective and interpersonal processes are closely involved in how rewarding music is experienced early in life. Because empathy is a well-established precursor of prosocial understanding and behavior, these findings further suggest that individual differences in how rewarding music feels may already be embedded in broader socio-emotional dispositions that support children's developing social orientation. Although the present study did not directly assess prosocial behavior, musical reward sensitivity may represent an early dispositional correlate of the broader links between music and social and interpersonal functioning across development.

By establishing an age-appropriate measurement framework and situating musical reward within a broader socio-emotional context, the present study lays the groundwork for future longitudinal investigations into how musical pleasure develops and how it may contribute to emotional and social functioning across development. More broadly, the findings suggest that, in middle childhood, music may represent a meaningful socio-emotional resource in educational settings, and not solely a domain for the development of technical or performance-based skills.

## Data Availability

The raw data supporting the conclusions of this article will be made available by the authors, without undue reservation.
