# Supplementary material for: Empathy and musical reward sensitivity in middle childhood: a psychometric validation of the Barcelona music reward questionnaire in children aged 7–9
Source: Front Cognit. 2026 Aug 13;5:1804916. doi: 10.3389/fcogn.2026.1804916 (PMC13518152; doi:10.3389/fcogn.2026.1804916)
Supplement: Supplementary file 1 [file Table_1.docx]

| **Item number** | **Italian item** | **English item** | **Subscale** | **Included in final 4-factor model** |
| --- | --- | --- | --- | --- |
| 1 | Quando condivido la musica con qualcuno, sento un legame speciale con quella persona | When I share music with someone I feel a special connection with that person | ScR | Yes |
| 2 | Nel tempo libero non ascolto quasi mai musica | In my free time I hardly listen to music | MS | No |
| 3 | Mi piace ascoltare musica che contiene emozioni | I like to listen music that contains emotion | EmE | Yes |
| 4 | La musica mi fa compagnia quando sono solo | Music keeps me company when I’m alone | MdR | Yes |
| 5 | Non mi piace ballare, nemmeno con la musica che mi piace | I don’t like to dance, not even with music I like | SeM | Yes |
| 6 | La musica mi fa essere vicino alle altre persone | Music makes me bond with other people | ScR | Yes |
| 7 | Mi emoziono quando ascolto certe musiche | I get emotional listening to certain pieces of music | EmE | Yes |
| 8 | La musica mi calma e mi rilassa | Music calms and relaxes me | MdR | Yes |
| 9 | La musica spesso mi fa ballare | Music often makes me dance | SeM | Yes |
| 10 | Sono sempre alla ricerca di nuova musica | I’m always looking for new music | MS | No |
| 11 | Mi possono venire le lacrime o da piangere quando ascolto una musica che mi piace tanto | I can become tearful or cry when I listen to a melody that I like very much | EmE | Yes |
| 12 | Mi piace cantare o suonare uno strumento con altre persone | I like to sing or play an instrument with other people | ScR | Yes |
| 13 | La musica mi aiuta a rilassarmi | Music helps me chill out | MdR | Yes |
| 14 | Non posso fare a meno di canticchiare o cantare la musica che mi piace | I can’t help humming or singing along to music that I like | SeM | Yes |
| 15 | A un concerto mi sento vicino ai musicisti e agli altri | At a concert I feel connected to the performers and the audience | ScR | Yes |
| 16 | Io e i miei genitori spendiamo molti soldi per la musica e strumenti musicali | I spend quite a bit of money on music and related items | MS | No |
| 17 | A volte mi vengono i brividi quando sento una melodia che mi piace | I sometimes feel chills when I hear a melody that I like | EmE | Yes |
| 18 | La musica mi consola | Music comforts me | MdR | Yes |
| 19 | Quando sento una musica che mi piace molto, non posso fare a meno di battere il tempo o muovermi a ritmo | When I hear a tune I like a lot I can’t help tapping or moving to its beat | SeM | Yes |

**Table S1. Full list of BMRQ-MC items in Italian and English, corresponding subscales, and inclusion in the final four-factor model**

Note: ScR = Social Reward; EmE = Emotional Evocation; MdR = Mood Regulation; SeM = Sensory-Motor; MS = Musical Seeking.
